# Relationship obsessive compulsive disorder: The hidden struggle in romantic relationships

**DOI:** 10.1192/j.eurpsy.2025.1714

**Published:** 2025-08-26

**Authors:** A. Karmous, H. Ktari, H. Ghabi, A. Hajri, E. Khelifa, A. Maamri, H. Zalila

**Affiliations:** 1 Emergency and outpatient department, Razi hospital, Manouba; 2 Psychiatry department, Tahar Maamouri hospital, Nabeul; 3 Department G, Razi hospital, Manouba, Tunisia

## Abstract

**Introduction:**

Obsessive-compulsive disorder (OCD) is a psychiatric disorder that presents a wide variety of clinical features and obsessional themes. A particular form of OCD has received growing attention from researchers and clinicians. Called relationship OCD (ROCD), this form concerns obsessive-compulsive symptoms arising around the romantic relationship and the partner.

**Objectives:**

The aim of this work was to explore ROCD through a case report and a systematic review.

**Methods:**

We report the case of a patient who visited the outpatient department of the Razi hospital (Tunisia) for obsessive thoughts evolving over 11 months. Moreover, a systematic review was conducted. PubMed via Medline, Google Scholar and Semantic Scholar were used as search engines. The keywords used were « Relationship obsessive compulsive disorder » or « ROCD » or « Relationship centered obsessive compulsive symptoms » or « Partner focused obsessive compulsive symptoms ». The publication period of the articles searched was from inception to December 2023. The language of the articles searched was either English or French.

**Results:**

Mr M.A, 25 years old, was in a relationship with a girl since two years. He was repeatedly wondering if he was in the right relationship and if he would be happier with another girl. He often sought reassurance from his friends and visualized photos of his girlfriend to ensure his feelings for her and to reduce these thoughts. The diagnosis of ROCD had been made. Cognitive behavior therapy sessions were carried out with the patient, with a clear decrease in obsessive thoughts and compulsive behaviors of checking and seeking reassurance.

The systematic review performed identified a total of 14 studies that were included in the final analysis. The mean age of patients was 30 years, with extremes of 19 and 84 years. In all the reviewed studies, ROCD symptoms were assessed using the “ROCD Inventory” and the “Partner-Focused Obsessive-Compulsive Inventory”. Study results suggest that obsessive symptoms linked to romantic relationships negatively affect the functioning of patients. Several cognitive distortions have been identified such as perfectionism and intolerance of uncertainty. Cognitive-behavioral therapy was the most used therapy in ROCD with a better understanding of one’s feelings and an improved decision-making ability.

**Conclusions:**

ROCD is a particular form of OCD that impacts on interpersonal relationships, particularly romantic or marital ones. A better understanding of this pathology by therapists would enable partners to overcome challenges and maintain healthy and fulfilling relationships.

**Disclosure of Interest:**

None Declared

